# Angiogenin ameliorates corneal opacity and neovascularization via regulating immune response in corneal fibroblasts

**DOI:** 10.1186/s12886-016-0235-z

**Published:** 2016-05-17

**Authors:** Seung Hoon Lee, Kyoung Woo Kim, Kwangsic Joo, Jae Chan Kim

**Affiliations:** Department of Ophthalmology, College of Medicine, Chung-Ang University Hospital, 224-1, Heukseok-dong, Dongjak-Gu, Seoul, 156-755 Republic of Korea; Graduate School of Chung-Ang University, College of Medicine, Seoul, Republic of Korea; Graduate School of Medical Science and Engineering, Korea Advanced Institute of Science and Technology, Daejeon, 305-701 Republic of Korea

**Keywords:** Angiogenin, Inflammation,IKK-ε

## Abstract

**Background:**

Angiogenin (ANG), a component of tears, is involved in the innate immune system and is related with inflammatory disease. We investigated whether ANG has an immune modulatory function in human corneal fibroblasts (HCFs).

**Methods:**

HCFs were cultured from excised corneal tissues. The gene or protein expression levels of interleukin (IL)-1beta (β), IL-4, IL-6, IL-8, IL-10, complements, toll-like receptor (TLR)4, myeloid differentiation primary response gene (MYD)88, TANK-binding kinase (TBK)1, IkappaB kinase-epsilon (IKK-ε) and nuclear factor-kappaB (NF-κB) were analyzed with or without ANG treatment in tumor necrosis factor-alpha (TNF-α)- or lipopolysaccharide (LPS)-induced inflammatory HCFs by real-time polymerase chain reaction (PCR), Western blotting and immunocytochemistry. Inflammatory cytokine profiles with or without ANG were evaluated through immunodot blot analysis in inflammatory HCFs. Corneal neovascularization and opacity in a rat model of corneal alkali burn were evaluated after application of ANG eye drops.

**Results:**

ANG decreased the mRNA levels of IL-1β, IL-6, IL-8, TNF-α receptor (TNFR)1, 2, TLR4, MYD88, and complement components except for C1r and C1s and elevated the mRNA expression of IL-4 and IL-10. Increased signal intensity of IL-6, IL-8 and monocyte chemotactic protein (MCP)-1 and MCP-2 induced by TNF-α or LPS was weakened by ANG treatment. ANG reduced the protein levels of IKK-ε by either TNF-α and LPS, and decreased TBK1 production induced by TNF-α, but not induced by LPS. The expression of NF-κB in the nuclei was decreased after ANG treatment. ANG application lowered corneal neovascularization and opacity in rats compared to controls.

**Conclusion:**

These results demonstrate that ANG reduces the inflammatory response induced by TNF-α or LPS in HCFs through common suppression of IKK-ε-mediated activation of NF-κB. This may support the targeting of immune-mediated corneal inflammation by using ANG.

## Background

The cornea, the transparent part of the eye, performs a significant function in eyesight by refracting the light to focus a visual image. As the cornea is indispensable for vision, corneal inflammation may induce visual disturbance and blindness. Several investigations have reported that various corneal inflammatory diseases cause visual impairment and chronic inflammation of the cornea can lead to blindness [1, 2]. It is well documented that chronic inflammation of human corneal fibroblasts (HCFs) leads to several corneal diseases including corneal opacity and ulceration, and these conditions lead to vision impairment in severe cases [3].

Although the human cornea is an immune privileged site in the body, chronic immune mediated inflammation, such as chemical burns or herpetic stromal keratitis, occasionally occurs in the corneal stroma, and HCFs residing in the corneal stroma conduct an important role in the regulation of local immune and inflammatory response [4]. HCFs also regulate the production of cytokines and play a role in the control of stromal inflammation [5, 6]. It has been reported that HCFs secrete pro-inflammatory cytokines and chemokines including interleukin (IL)-6 and IL-8 in response to inflammatory stimuli [4, 5, 7]. Moreover, HCFs modulate complement activation in diverse immune responses. For instance, in a healthy human eye, HCFs synthesize complement components and the complement cascade is activated chronically at a low level, but complement component 3 (C3) and C5 production is up-regulated by several cytokines [8, 9]. The complement system is an important part of innate immunity and its activation plays a major role in the inflammatory response. It has been widely accepted that complement activation causes inflammation via nuclear translocation of nuclear factor-kappaB (NF-κB) [10]. The activation of the complement system in the normal human eyes is initiated by the immune response and it induces advancement of corneal inflammation [11]. As one of the mechanisms, it has been suggested that complement activation is triggered by inflammatory stimuli such as tumor necrosis factor-alpha (TNF-α) or lipopolysaccharide (LPS) [12, 13].

TNF-α is a cytokine that induces inflammatory responses and binds to two receptors, TNF-α receptor (TNFR)1 and TNFR2. It has been demonstrated that TNF-α plays a critical role in corneal inflammation [14]. LPS released from gram-negative bacteria induces innate immune response by binding to the toll like receptor (TLR)4 and the activation of TLR4 causes pro-inflammatory cytokines production such as IL-6 and IL-8 in the cornea [15]. It is well known that LPS provokes inflammation in the cornea [16]. TNF-α and LPS promote the nuclear translocation of NF-κB and activate NF-κB signaling pathway, which is indispensable in all mammalian cell types and controls several genes involved in immune-inflammatory responses [17–19].

The nuclear translocation of NF-κB related to the inflammatory response is inhibited by regulatory protein IkappaB (IκB), which normally prevents nuclear translocation of NF-κB by arresting it in the cytoplasm [20]. In the activation of NF-κB and the downstream transcription, IκB kinase epsilon (IKK-ε) and TANK-binding kinase (TBK)1 are known as the promoters [21, 22]. The activation of IKK-ε causes secretion of pro-inflammatory cytokines and the sequence of IKK-ε showed 49 % homology with the sequence of TBK1, which mediates NF-κB signaling pathway in inflamed HCFs [23, 24]. TNF-α and LPS cause activation of IKK-ε and TBK1 by inducing inflammation through the nuclear translocation of NF-κB, in contrast, an inhibitor of IKK-ε and TBK1 has the potential to decrease inflammation [20, 25].

Angiogenin (ANG) is a 14.4 kDa single chain protein containing 123 amino acids and the expression of ANG is associated with an inflammatory response. It has been reported that ANG levels were increased in serum of inflammatory bowel disease patients [26]. The mRNA expression and protein levels of ANG are increased in an inflammatory environment such as treatment with TNF-α and IL-1β [27, 28]. Previous evidences have been demonstrated that ANG has the potential to influence innate immune modulation. It has long been recognized that ANG is a microbial protein involved in innate immunity and has bactericidal activity [29]. ANG is also known as a component of the tear fluid and plays an important role in protecting the ocular surface as it is an antimicrobial peptide [30]. In our previous report, it was well documented that ANG down-regulates pro-inflammatory cytokines expression through inhibition of TBK1 production in HCFs [24].

The present study was performed to investigate the anti-inflammatory activity of ANG in the cornea. First, we investigated the anti-inflammatory effect of ANG using a rat model of corneal alkali burn in vivo. Second, we studied the anti-inflammatory activity of ANG, which inhibits the expression of pro-inflammatory cytokines, chemokines and complement components during the corneal inflammatory process. Finally, in order to understand how ANG reduces the inflammatory response, we undertook to elucidate its effect on the NF-κB pathway mediated by IKK-ε in TNF-α- or LPS-induced inflammatory HCFs.

## Methods

### Animal model for corneal alkali burn

Twenty healthy adult male Sprague Dawley rats (weight range, approximately 250–270 g) were selected for this investigation. The animals were initially examined and screened for any preexisting ophthalmic lesions. The rats were anesthetized via intramuscular injection of 0.05 cc/50 g of tiletamine plus zolazepam (Zoletil™, Virbac, Fort Worth, TX, USA) and 0.05 cc/100 g of xylazine (Rompun™, Bayer, Leverkusen, Germany). Alkali burn was induced in the right eye by 60 s of exposure of the corneal periphery including limbus to a 4 mm diameter disk of filter paper soaked in 1 N NaOH rinsing with sterile saline. The control group (*n* = 10) was treated topically with 10 μl of PBS, and the treatment group (*n* = 10) was treated with 10 μl of ANG (50 μg/ml) eye drops four times a day immediately after the alkali injury. The treatments were administered daily at the same time for a total period of 56 days.

### Evaluation of corneal opacity and neovascularization

The corneal opacity was measured using the scoring system [31] to evaluate the degree of corneal opacification between 0 to +4. The area of corneal neovascularization was quantified from the photographs based on the ratio of total corneal area using Image J software and previously established formula such as A = C/12 x 3.1416 [*r*^*2*^*- (r -l)*^*2*^] (A : The area of corneal neovascularization, C : the number of clock hour, *r* : Radius of rat cornea, *l* : Reticule the vessel length) [32]. The mean differences in corneal opacities and corneal NV area were compared between the control group and ANG treatment group.

### Isolation and primary culture of human corneal fibroblast cells

Human corneal donor tissues were obtained during penetrating keratoplasty. The method of isolation and primary culture of HCFs was described in a previous article [24]. In briefly, the corneal epithelium was eliminated and then the corneal fibroblast cells were detached from the explant tissue. The corneal tissues were rinsed with phosphate-buffered saline (PBS) mixed with 5 % penicillin-streptomycin and cut into explants of approximately 1 mm^3^. The HCFs were then subcultured by trypsin digestion and cultured in alpha-minimum essential medium (α-MEM) (Invitrogen, Waltham, MA, USA) containing 10 % FBS and 1 % penicillin-streptomycin. The cells were maintained at 37 °C under 5 % CO 2 and used for experiments after three to five passages.

### Cell treatment

HCFs were cultured in six-well plates for 3 days. The cells were washed twice with PBS. The medium containing the confluent corneal fibroblast cells was changed to serum-free MEM for 1 day before treatment. The cells were treated with TNF-α (20 ng/ml) purchased from ProSpec (Ness-Ziona, Israel) for eight hours or LPS (1 μg/ml) purchased from Sigma-Aldrich (St. Louis, MO, USA) for four hours, and with or without ANG (2 μg/ml) for 30 min. ANG was obtained from the Department of Biochemistry at Chungbuk National University in Korea and the identity of the purified ANG was confirmed by Western blotting with ANG-specific antibodies with methods described in a previous report [33]. The biological activity of the purified ANG also was confirmed by its nuclear translocation in human umbilical vein endothelial cells by a procedure previously described in detail [33]. The purification and endotoxin levels of recombinant ANG expressed in E. coli are described previously [24]. The cells were then collected for total RNA isolation and protein extraction.

### RNA isolation and real-time RT-PCR

Total RNA was isolated from cultured HCFs using rat corneal tissue and FavorPrep™ Tri-RNA reagent (Favorgen Biotech Corp., Ping-Tung, Taiwan) according to the manufacturer’s protocols. The quantity and quality of the RNA was determined using a NanoDrop ND-1000 spectrophotometer (Nano-Drop Technologies, Inc. Wilmington, DE, USA). Single-stranded complementary DNA (cDNA) was synthesized from 500 ng of total RNA using a cDNA synthesis kit (Takara Bio, Inc., Otsu, Japan). Real-time RT-PCR was conducted using the CFX96TM Real-Time PCR Detection System (Bio-Rad Laboratories, Inc., Hercules, CA, USA) in a total volume of 20 μL containing 10 μL of SYBR Premix Ex Taq (Takara Bio, Inc.), diluted cDNA template, and forward and reverse primers. The primer sequences and product sizes are listed in Table 1. PCR amplification for the selected genes was run for 40 cycles. Gene expression was analyzed by real-time reverse transcriptase polymerase chain reaction (PCR). Real-time PCR quantification was performed in triplicate for each sample and the mean was calculated. Expression levels were analyzed by real-time PCR using values of glyceraldehyde-3-phosphate dehydrogenase (GAPDH) as a reference.

**Table 1:** Sequences of PCR primers

Gene	Sense primer (5’→3’)	Anti-sense primer (3’→5’)	Product size (bp)
*GAPDH*	CGAGATCCCTCCAAAATCAA	TGTGGTCATGAGTCCTTCCA	294
*IL-1*β	CCTGTCCTGCGTGTTGAAAGA	GGGAACTGGGCAGACTCAAA	150
*IL-4*	AACACAACTGAGAAGGAAACCTTC	GCTCGAACACTTTGAATATTTCTC	276
*IL-6*	TTCGGTCCAGTTGCCTTCTC	GAGGTGAGTGGCTCTCTGTG	112
*IL-8*	ACATGACTTCCAAGCTGGCCG	TTTATGAATTCTCAGCCCTC	302
*IL-10*	GCCTAACATGCTTCGAGATC	TGATGTCTGGGTCTTGGTTC	206
*TLR4*	AGCCTAAGCCACCTCTCTACCT	AGATTTGTCTCCACAGCCACCA	116
*MYD88*	ATGGTGGTGGTTGTCTCTGATG	GACAGGATGAACCTCAGGATGC	165
*TNFR1*	GTGCTGTTGCCCCTGGTCAT	GCTTAGTAGTAGTTCCTTCA	163
*TNFR2*	AAACTCAAGCCTGCACTC	GGATGAAGTCGTGTTGGAGA	209
*C1r*	CAACAACTTTGAAACAACCA	GGAGAAGTCTGTGTGGAAGG	237
*C1s*	AAGTCAGACTTTTCCAATGA	ACTTGCAATCTCCCCAATCA	247
*C2*	TGGAGTGGACAAGCTGTGCCG	TGAAAGTCTCGTGGCGGCGG	289
*C3*	TGGCTGTTCGCACCCTGGAT	AGCCCGAGGGGGTCACAATGA	204
*C4*	ATGGTTCCTATGCGGCTTGGTTGTC	GCGATGGTCACAAAGGCTGTGAGTG	256
*C5*	TGGCAACCAGCTCCAGGTTCAT	TGCCCCACAGCCCAGATCACT	208
*CFB*	CAAGAAGGCCCTCCAGGCAGT	AAGGCCCGACCCCAAACACAT	233
*TBK1*	TTCTGGAAGTCCATACGCAT	ACTGGTGATCTCTATGCTGT	237
*IKK-*ε	CTGCTCATGAATGACAGTGA	GGCGAGTGTATGTTATGCTT	132

### Immunodot blot assay

The expression of 42 human cytokines and chemokines was assessed using a commercially available cytokine assay (RayBio Human Cytokine Antibody Array three, RayBiotech, Norcross, GA, USA) that utilizes membrane-bound cytokine-specific antibodies to assess for the presence of several cytokines in biological fluids. The analysis was conducted according to the manufacturer’s instructions. Briefly, membranes were blocked for 30 min and then incubated with HCF culture supernatant for two hours at room temperature. The membranes were washed with Wash Buffer I three times for 5 min each and then with Wash Buffer II twice for 5 min each. After washing, the membranes were incubated with a biotin-conjugated antibody mix for two hours, and then streptavidin-conjugated peroxidase was added for two hours at room temperature. The membranes were subsequently washed thoroughly and exposed to chemiluminescence. The membranes were visualized using the ECL Plus detection system and ChemiDocTM XRS (Bio-Rad Laboratories Inc.). The densities for individual spots were calculated using Image J software (National Institutes of Health, USA). The relative expression ratio was determined by subtraction of the background signal and comparison with positive controls on the membrane. Positive controls visible within each array were used for comparison.

### Nuclear and cytosolic protein extractions

HCFs were washed and scraped with cold PBS. The cells were lysed in buffer A (10 mM HEPES [pH 7.9], 10 mM KCl, 0.1 mM EDTA, 0.1 mM EGTA, 1 mM DTT, 0.5 mM PMSF, 5 μg/ml Leupeptin) and left on ice for 15 min. After 10 % NP-40 was added to the sample, the cytosolic fraction was collected by centrifugation at 14,000 rpm for 5 min at 4 °C. The nuclear fraction was re-suspended in buffer C (20 mM HEPES [pH7.9], 0.4 n NaCl, 1 mM EDTA, 1 mM EGTA, 1 mM DTT, 1 mM PMSF, 10 μg/ml Leupeptin) and left on ice for 30 min, and then the nuclear fraction was collected by centrifugation at 14,000 rpm for 5 min at 4 °C.

### Western blot analysis

Total cellular protein was isolated from cultured cells with a protein extraction solution (PRO-PREP, iNtRON biotechnology, Inc., Seongnam-si, Gyeonggi-do, Korea). Nuclear proteins and total cell lysates were separated by 10 % sodium dodecyl sulfate polyacrylamide gel electrophoresis (SDS-PAGE) and electrophoretically transferred to a polyvinylidene fluoride membrane (PVDF; Merck Millipore, Billerica, MA, USA) at 100 V (1 h, 4 °C) in buffer containing 0.3 % Tris, 1.4 % glycine, and 20 % methanol using a wet-blotting apparatus (Mini-PROTEANTetra cell; Bio-Rad Laboratories, Inc.). The PVDF membrane containing the transferred proteins was blocked with 5 % BSA in PBS for one hour at room temperature. Primary monoclonal antibodies against human IKK-ε (Abcam, Cambridge, MA, USA), TBK1 (Abcam), and NF-κB (Bioworld Technology, Inc., St. Louis, MN, USA) diluted in PBS (1:1000) was applied to the PVDF membrane and incubated overnight at 4 °C. Secondary antibodies diluted in PBS (1:2000) were subsequently applied to the PVDF membrane and incubated for 1 h at room temperature. The PVDF membrane was washed four times (10 min each) with Tris-buffered saline (TBS; 50 mM Tris HCl pH 7.5, 150 mM NaCl) containing 0.1 % Tween 20. The binding of specific antibodies was visualized using an enhanced chemiluminescence Western blotting detection kit (Pierce Biotechnology, Inc., Rockford, IL, USA). Densitometric quantification of the immunoblot was carried out using ImageJ software. The value of each band was normalized to β-actin or lamin.

### Immunocytochemistry

The HCFs cultured on glass slides were treated with TNF-α (20 ng/ml) for eight hours or with LPS purchased from Sigma-Aldrich (1 μg/ml) for four hours. Cells were also treated with or without ANG (2 μg/ml) for 30 min. Cells were then fixed in 4 % paraformaldehyde for 15 min at room temperature. After being permeabilized by incubation with 0.5 % Triton X-100 for 15 min at room temperature, the slides were incubated with anti-NF-κB (diluted to 1:50 in PBS, Bioworld Technology, Inc.) for 1 h at room temperature. Glass slides were incubated with secondary antibody for 1 h at room temperature. At each step, the slides were washed three times (5 min each) with PBS. Cover slips were mounted on the slides using Vectashield (Vector Laboratories, Burlingame, CA, USA) containing 40,6-diamidino-2-phenylindole (DAPI).

### Statistical analysis

Data are expressed as the mean ± standard error (SE). Statistical analysis of three separate experiments was conducted using one-way ANOVA followed by a *post hoc* pairwise comparison adjusted with a Bonferroni correction and analysis of two separate was performed using Mann–Whitney *U* test. Statistical analyses were performed using the SPSS software version 19.0 (SPSS Inc., Chicago, IL, USA). Differences were considered statistically significant at *p* < 0.05.

## Results

### ANG improves corneal inflammation in in vivo rat models with alkali burn

**Fig. 1:**
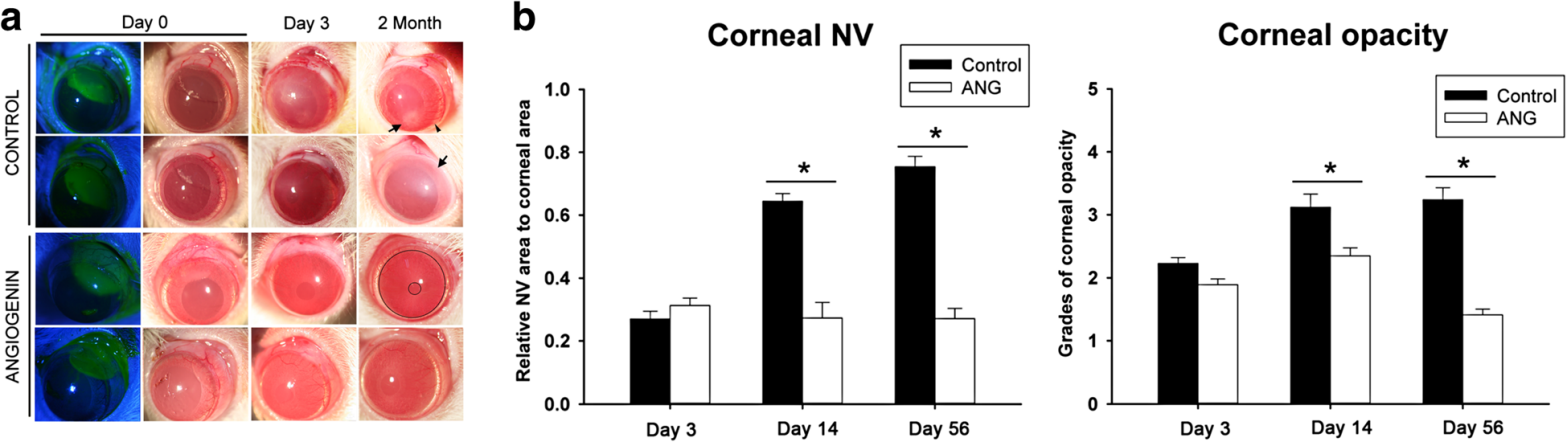
Comparison of corneal opacity and neovascularization in rat corneas with alkali burn between the angiogenin (ANG) group and the control group. **a** Representative photographs demonstrated the corneal surface on day 3 and 56 after injury. Marked corneal opacity (*arrow*) was noted by day 56 after injury, and significant neovascularization (*arrow head*) developed by day 56 after injury to the cornea in the control group. In contrast, the clear transparent cornea (*inside large black circle*) and clear papillary margin (*small black circle*) in the ANG-treated cornea is noted. **b** Quantification of corneal opacity following a clinical grading system on a scale from 0 to +4 and quantification of corneal neovascularization. Corneal opacity and neovascularization were significantly down-regulated by ANG administration. Quantified estimates of corneal neovascularization are expressed as relative ratio of the neovascularizaed area to the whole corneal area. Values represent the mean ± standard error. Mann–Whitney *U* test, *p < 0.05

ANG-treated eyes of rat models almost recovered their normal corneal transparency and showed a significant reduction in the corneal opacity score compared to the control eyes at 14 days after alkali injury. Until 2 months after alkali burn in rat models, ANG eye drops significantly improved the signs of alkali-induced representative corneal inflammation such as corneal opacity and neovascularization at the peripheral cornea, including conjunctiva compared to that in controls (Fig. 1a and b). Corneal epithelial erosions were recovered at 3 days and did not recur until 2 month-follow up in both control and ANG-treated groups.

### ANG inhibits pro-inflammatory cytokines and enhances anti-inflammatory cytokines in HCFs

**Fig. 2:**
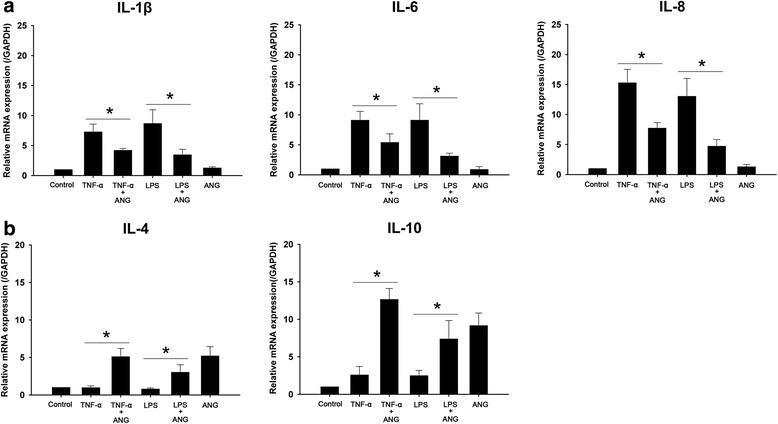
Real-time PCR analyses of pro-inflammatory and anti-inflammatory cytokines in human corneal fibroblasts (HCFs) with or without angiogenin (ANG) treatment. **a** The increased relative mRNA levels of IL-1β, IL-6 and IL-8 by tumor necrosis factor-alpha (TNF-α) or lipopolysaccharide (LPS) were diminished by ANG treatment. **b** The relative mRNA levels of IL-4 and IL-10 were increased by ANG treatment. The experiments were repeated at least three times. Values represent the mean ± standard error. One-way ANOVA followed by Bonferroni’s *post hoc* analysis, *p < 0.05

In order to determine whether ANG can reduce the inflammatory responses in HCFs, TNF-α or LPS was added to the culture media to induce inflammation and then cells were cultured in the presence or absence of ANG. Real-time PCR was performed to investigate the effects of ANG treatment on the mRNA expression of pro-inflammatory (IL-1β, IL-6 and IL-8) and anti-inflammatory cytokines (IL-4 and IL-10). The mRNA expression of IL-1β, IL-6 and IL-8 induced by TNF-α or LPS treatment was decreased significantly in HCFs when treated with ANG (Fig. 2a). Moreover, the mRNA expression of anti-inflammatory cytokines (IL-4 and IL-10) was increased significantly after ANG treatment (Fig. 2b).

**Fig. 3:**
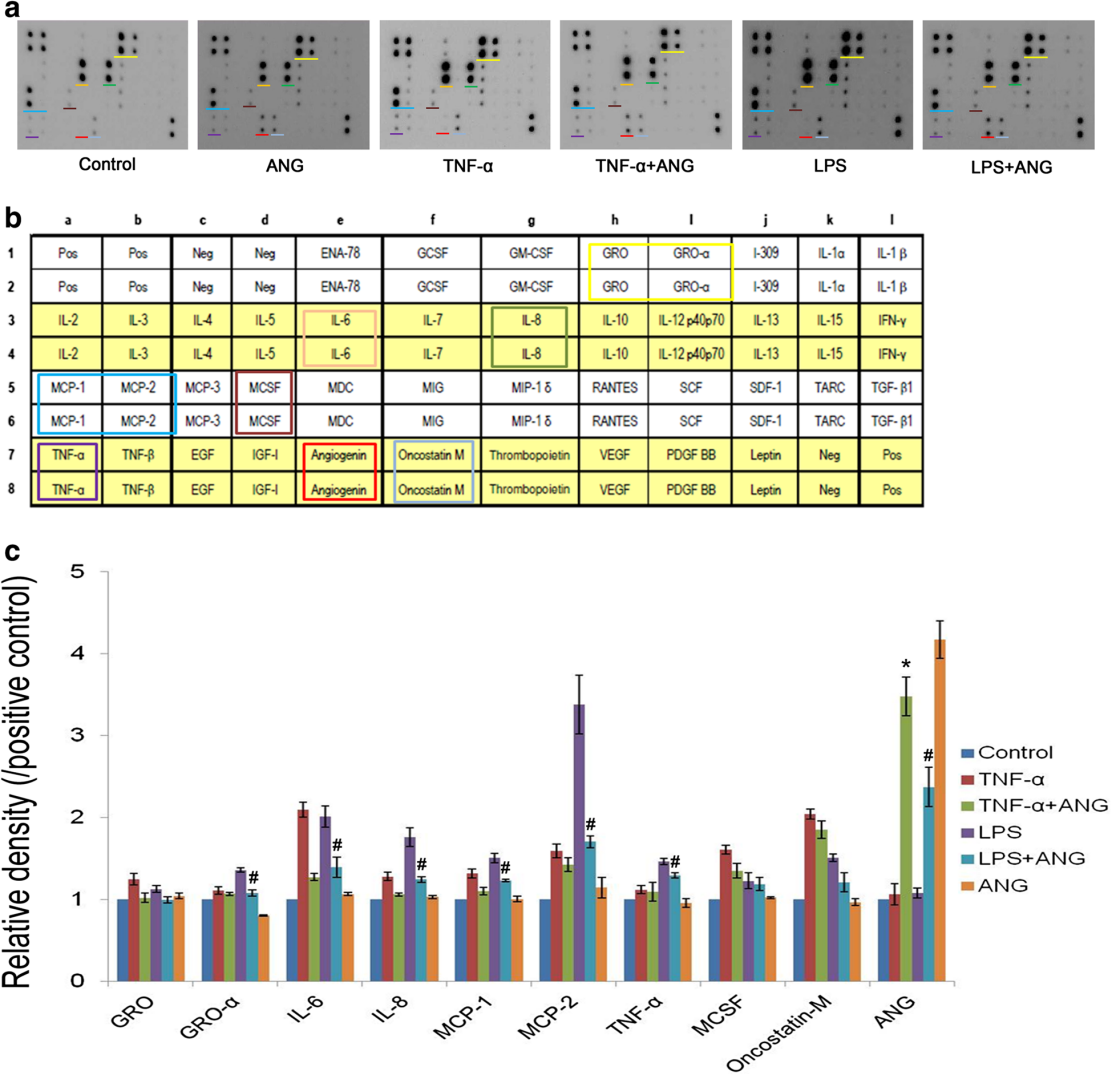
Immunodot blot analysis of inflammatory cytokine profiles in culture medium of human corneal fibroblasts (HCFs) with or without angiogenin (ANG) treatment. **a** Treatment of HCFs with tumor necrosis factor-alpha (TNF-α, 20 ng/ml) resulted in amplification of five inflammatory cytokines and chemokines including growth regulated oncogene (GRO), TNF-α, interleukin (IL)-6, IL-8, and monocyte chemotactic protein (MCP)-1. Treatment of HCFs with lipopolysaccharide (LPS, 1 μg/ml) resulted in amplification of five inflammatory cytokines and chemokines including GRO, TNF-α, IL-6, IL-8, and MCP-1. Treatment of HCFs with ANG (2 μg/ml) resulted in reduction in expression of these inflammatory cytokines and chemokines. **b** Cytokine Antibody Array Map. Pos: positive control; Neg: negative control; ENA: epithelial neutrophil-activating protein; GCSF: granulocyte colony-stimulation factor; GM-CSF: granulocyte macrophage CSF; IFN-γ: interferon-gamma; MCSF: macrophage CSF; MDC: monocyte chemotactic protein; MIG: monokine induced by IFN-γ; MIP: macrophage inflammatory protein; RANTES: Regulated on Activation, Normal T Cell Expressed and Secreted; SCF: stem cell factor; SDF-1: stromal cell derived factor-1; TARC: thymus and activation-regulated chemokine; TGF-β1: transforming growth factor-beta1; EGF: epidermal growth factor; IGF-1: insulin-like growth factor; VEGF: vascular endothelial growth factor; PDGF: platelet-derived growth factor. **c** Relative density of inflammatory cytokines and chemokines. The values presented in the bar graph are the mean ± standard error from triplicate experiments. One-way ANOVA followed by Bonferroni’s *post hoc* analysis, *p < 0.05 versus TNF-α without ANG; #*p* < 0.05 versus LPS without ANG

Immunodot blot assays were conducted to determine whether ANG reduces inflammatory cytokines and chemokines in media. Treatment with TNF-α or LPS promoted the expression of inflammatory cytokines and chemokines such as IL-6 and IL-8, growth-related proteins (GRO), GRO-α and monocyte chemotactic protein (MCP)-1, MCP-2, monocyte chemotactic protein (MCSF), oncostatin-M and TNF-α. Production of these cytokines and chemokines at protein levels was downregulated in the presence of ANG, but expression of ANG was self-up-regulated by ANG treatment (Fig. 3a and b). We detected significant differences when comparing media before and after ANG treatment with respect to the presence of IL-6, IL-8, MCP-1 and ANG in the medium obtained from TNF-α-treated HCFs. In the medium obtained from LPS-treated HCFs, ANG treatment significantly reduced the production of GRO-α, IL-6, IL-8, MCP-1, MCP-2 and TNF-α (Fig. 3c).

### ANG reduces the expression of TNFR, TLR4, MYD88 and complement components

**Fig. 4:**
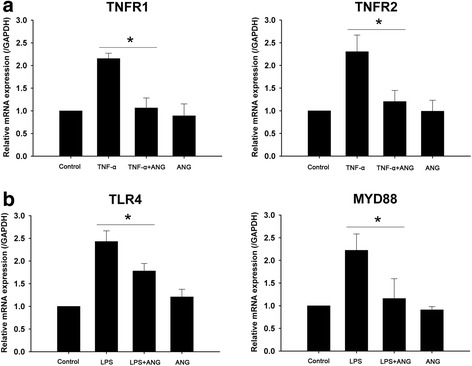
Real-time PCR analyses of tumor necrosis factor-alpha receptor (TNFR)1, TNFR2, toll-like receptor (TLR)4 and myeloid differentiation primary response gene (MYD)88 in human corneal fibroblasts (HCFs) with or without angiogenin (ANG) treatment. **a** The relative mRNA level of TNFR1, and TNFR2 was diminished by ANG treatment in HCFs stimulated with TNF-α. **b** The relative mRNA level of TLR4 and MYD88 was diminished by ANG treatment in HCFs stimulated with lipopolysaccharide (LPS). The experiments were repeated at least three times. One-way ANOVA followed by Bonferroni’s *post hoc* analysis, *p < 0.05

Using real-time PCR, we determined whether ANG decreases mRNA level of TNFR1, TNFR2, TLR4, MYD88 and complement components. TNF-α treatment up-regulated the mRNA expression of TNFR1 and TNFR2, but a significant reduction was noted after ANG treatment. ANG treatment also decreased TLR4 and MYD88 mRNA expression induced by LPS (Fig. 4).

**Fig. 5:**
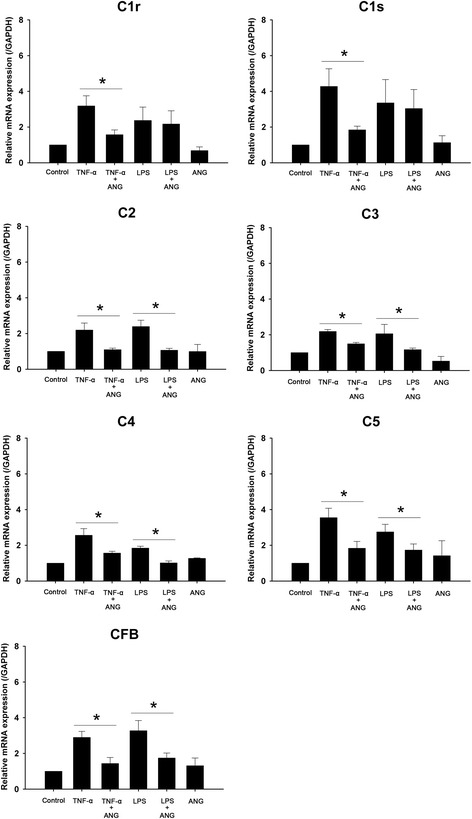
Real-time PCR analyses of complement components in human corneal fibroblast (HCF) cells with or without angiogenin (ANG) treatment. The relative mRNA level of complement components was diminished by ANG treatment in HCFs stimulated with tumor necrosis factor-alpha (TNF-α). Except for C1r and C1s, levels of all complements were reduced by ANG treatment in lipopolysaccharide (LPS)-stimulated HCFs. The experiments were repeated at least three times. One-way ANOVA followed by Bonferroni’s *post hoc* analysis, *p < 0.05

The mRNA expression of complement components was induced by TNF-α or LPS. After ANG treatment, the increased mRNA expression of complement components induced by TNF-α was reduced. All of the activated complements influenced by LPS except for C1r and C1s were inhibited by ANG at the mRNA level (Fig. 5).

### ANG reduces IKK-ε and TBK1 production in HCFs stimulated with LPS

**Fig. 6:**
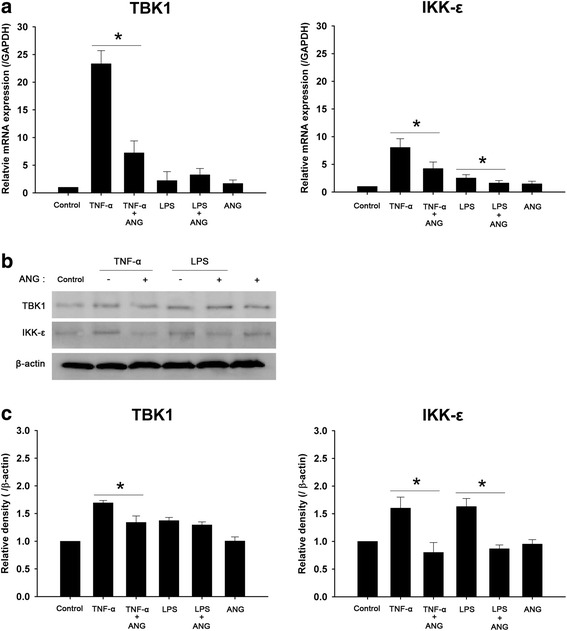
Real-time PCR analyses and Western blot analyses of TANK-binding kinase (TBK)1 and IkappaB kinase-epsilon (IKK-ε) in human corneal fibroblast (HCF) cells with or without angiogenin (ANG) treatment. **a** The relative mRNA level of TBK1 was decreased by ANG in HCFs stimulated with tumor necrosis factor-alpha (TNF-α), and not in those stimulated with lipopolysaccharide (LPS); and the relative mRNA level of IKK-ε was diminished by ANG in HCFs stimulated either with TNF-α or LPS. **b** ANG treatment reduced TBK1 protein expression in HCFs stimulated with TNF-α, and not in those stimulated with LPS, and down-regulated IKK-ε protein expression in HCFs stimulated either with TNF-α or LPS. **c** Densitometric analysis of the expression of TBK1 and IKK-ε protein relative to beta-actin in HCFs. The experiments were repeated at least three times. One-way ANOVA followed by Bonferroni’s *post hoc* analysis, *p < 0.05

The mRNA expression of IKK-ε and TBK1 was increased by TNF-α or LPS. The ANG decreased the mRNA expression of TBK1 and IKK-ε induced by TNF-α, and decreased the mRNA expression of only IKK-ε which was induced by LPS (Fig. 6a). According to Western blot analysis, TNF-α or LPS treatment increased IKK-ε and TBK1 expression in HCFs. TBK1 protein expression was down-regulated in HCFs stimulated with TNF-α, but not in those stimulated with LPS after ANG treatment on HCFs. IKK-ε expression was reduced by ANG treatment in HCFs stimulated with either TNF-α or LPS (Fig. 6b and c).

### ANG inhibits nuclear translocation of NF-κB in HCFs

**Fig. 7:**
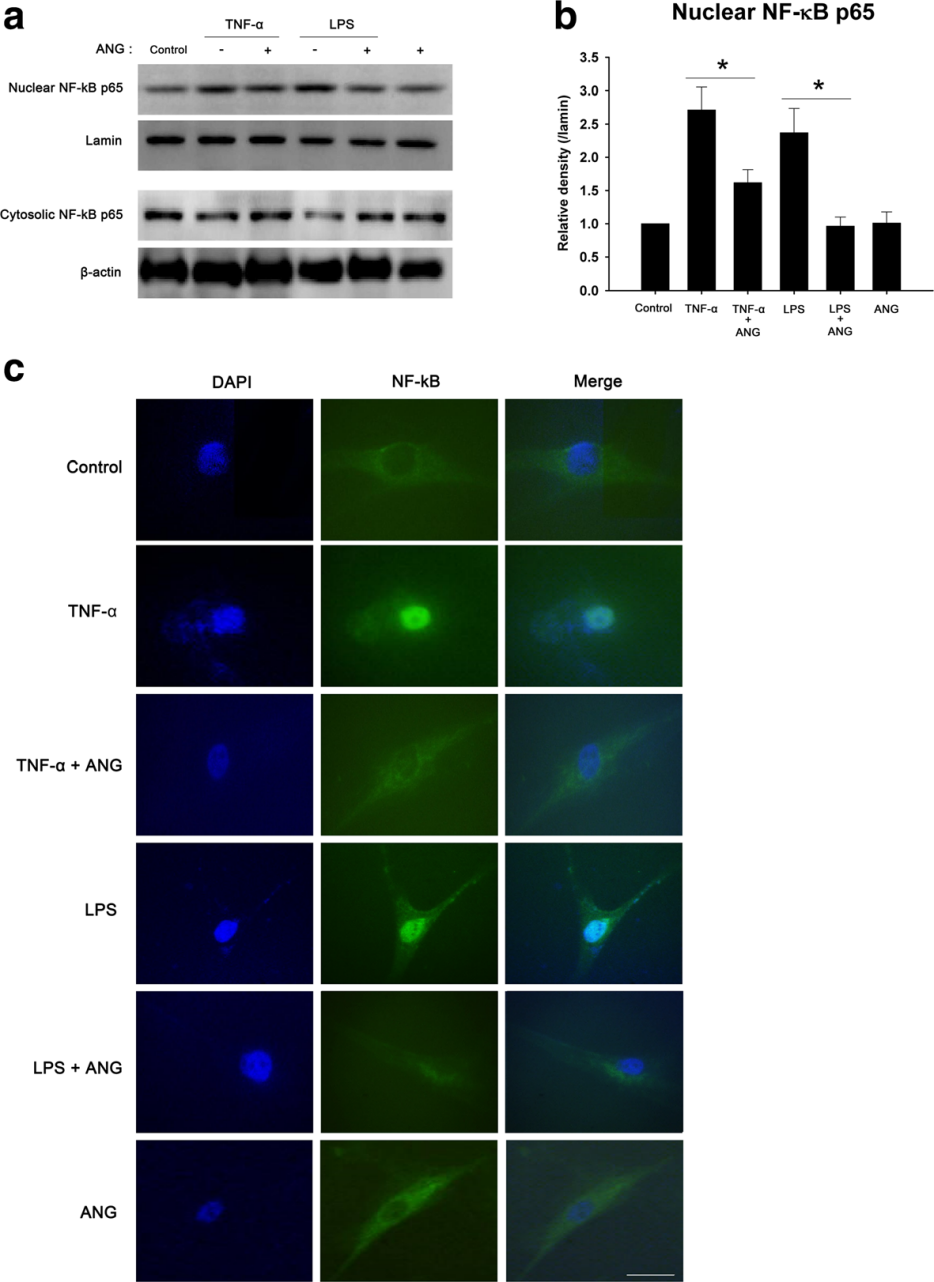
Western blot and immunocytochemical analyses of nuclear factor-kappaB (NF-κB) in the nucleus and cytoplasm of human corneal fibroblasts (HCFs) with or without angiogenin (ANG) treatment. **a** After ANG treatment, nuclear translocation of NF-κB induced by tumor necrosis factor-alpha (TNF-α) or lipopolysaccharide (LPS) was decreased. **b** Densitometric analysis of the nuclear expression of NF-κB protein relative to lamin in HCFs. The experiments were performed in triplicate. One-way ANOVA followed by Bonferroni’s *post hoc* analysis, *p < 0.05. **c** Immunocytochemistry of NF-κB in the nuclei and cytoplasm of HCFs. The intranuclear fluorescence of NF-κB induced by TNF-α or LPS was suppressed by ANG treatment. Scale bar, 100 μm

HCFs were cultured with either TNF-α or LPS and treated with ANG to examine whether ANG reduces nuclear translocation of NF-κB in HCFs. On Western blotting and immunofluorescent analysis, treatment of HCFs with TNF-α or LPS increased the translocation of NF-κB from the cytosol to the nucleus, on the contrary, ANG treatment reversed the nuclear translocation of NF-κB into the cytosol (Fig. 7).

## Discussion

ANG which has been demonstrated extensively as an angiogenic molecule [34], also has antimicrobial activity and is reported to be one of the tear components. This protein is also known to be related to inflammatory diseases and innate immunity. However, there is little mechanistic evidence of the effect of ANG on the inflammatory response. Here, we studied the anti-inflammatory activity of ANG on inflamed HCFs via previously undiscovered novel mechanisms.

The most meaningful finding of this study is that ANG down-regulated TNF-α or LPS-induced inflammatory response via suppression of IKK-ε expression. Several reports have showed that the activation of IKK-ε leads to inflammation mediated by NF-κB [35, 36]. Moreover, it has been demonstrated that the protein kinase IKK-ε regulates the expression of inflammatory cytokines such as IL-6, -8, MCP-1 and TNF-α [21, 37]. As the inhibition of IKK-ε expression is a well-known important property for reducing inflammation, our result indicating the anti-inflammatory function of ANG in inflamed HCFs through the reduction of IKK-ε production suggests a new perspective for inflammatory modulation in human corneas.

Scar formation and corneal opacity are important in corneal inflammation. It is well documented that scar formation is an abnormal state of wound healing suggesting an excessive activity of fibroblast cells during wound healing [38]. Moreover, corneal opacity is resulted in wound and downregulated by suppression of corneal inflammation [39, 40]. Our results reveal that ANG treatment reduced corneal opacity and scar formation at the peripheral cornea including conjunctiva. It can suggest that ANG has a potential to be applied to corneal inflammatory disorder clinically.

LPS generally leads to inflammation, inducing the release of pro-inflammatory cytokines and chemokines such as TNF-α, IL-6, IL-8, MCP-1 and MCP-2 [41, 42]. TNF-α also causes inflammatory responses producing several pro-inflammatory cytokines including IL-6, IL-8, and MCP-1 [43, 44]. It is well demonstrated that IL-6 and IL-8 perform a crucial role in initiating chronic inflammation and, additionally in corneal inflammatory diseases [45–48]. IL-4 and IL-10 known as the anti-inflammatory cytokines restrain the immune response and inhibit the expression of pro-inflammatory cytokines [49]. The noticeable finding in this study is that the expression of TNF-α, IL-6, IL-8, MCP-1 and MCP-2 induced by TNF-α or LPS was reduced after ANG treatment. In contrast, the mRNA expression of IL-4 and IL-10 was up-regulated by ANG treatment. On the basis of these results, we presumed that ANG may have anti-inflammatory function in suppression of pro-inflammatory cytokines such as TNF-α, IL-6, IL-8, MCP-1 and MCP-2 in HCFs.

Previously, several reports have suggested that IL-6 regulates ANG expression [50, 51]. But, interestingly, ANG treatment induces cytokine production including IL-6 in this study. It is well documented that ANG activates extracellular signal-regulated kinases (ERK) and causes angiogenesis and nitric oxide synthesis [52]. Thus, induction of several cytokines expression could have been occurred by ANG treatment.

NF-κB is a significant regulator of the immune response and inflammation. The activation of NF-κB elevated the expression of inflammatory cytokines and is related with several inflammatory diseases [53]. Inflammatory responses induced by LPS or TNF-α via activation of TLR4, MYD88, TNFR1 or TNFR2 cause NF-κB activation, which is suppressed by IκB proteins including TBK1 and IKK-ε, thus, isolating NF-κB in the cytoplasm [54]. The complement activation also causes nuclear translocation of NF-κB [10]. It has been proposed that TBK1 and IKK-ε conduct an essential role in inflammation and the inhibition of TBK1 and IKK-ε is a target for inflammatory diseases [25]. The inhibitor of complement activation has also been reported as an anti-inflammatory therapeutic compound [55]. It is well documented that the inhibitor of complement activation has the potential of a drug for inflammatory disease such as arthritis [56]. Our serial results suggest that ANG may inhibit NF-κB nuclear translocation through a decrease in TBK1 and IKK-ε production and inhibition of TLR4, MYD88, TNFR1, TNFR2, and complement component mRNA expression in TNF-α- or LPS-inflamed HCFs.

Corneal alkali burn results in an excessive immune response of the cornea inducing corneal opacity and neovascularization [57]. Because it was shown that ANG reduced corneal opacity and neovascularization in corneal alkali burn in this study, it is expected that ANG can suppress the immune-mediated inflammatory responses in chemical burns of the eye as verified in an in vitro analysis. Additionally, although ANG is also known as a stimulator of new vessel formation through the process of angiogenesis, ANG treatment did not cause any adverse effect such as injection on the ocular surface. We expect that ANG has the potential as a therapeutic molecule against ocular inflammatory diseases.

**Fig. 8:**
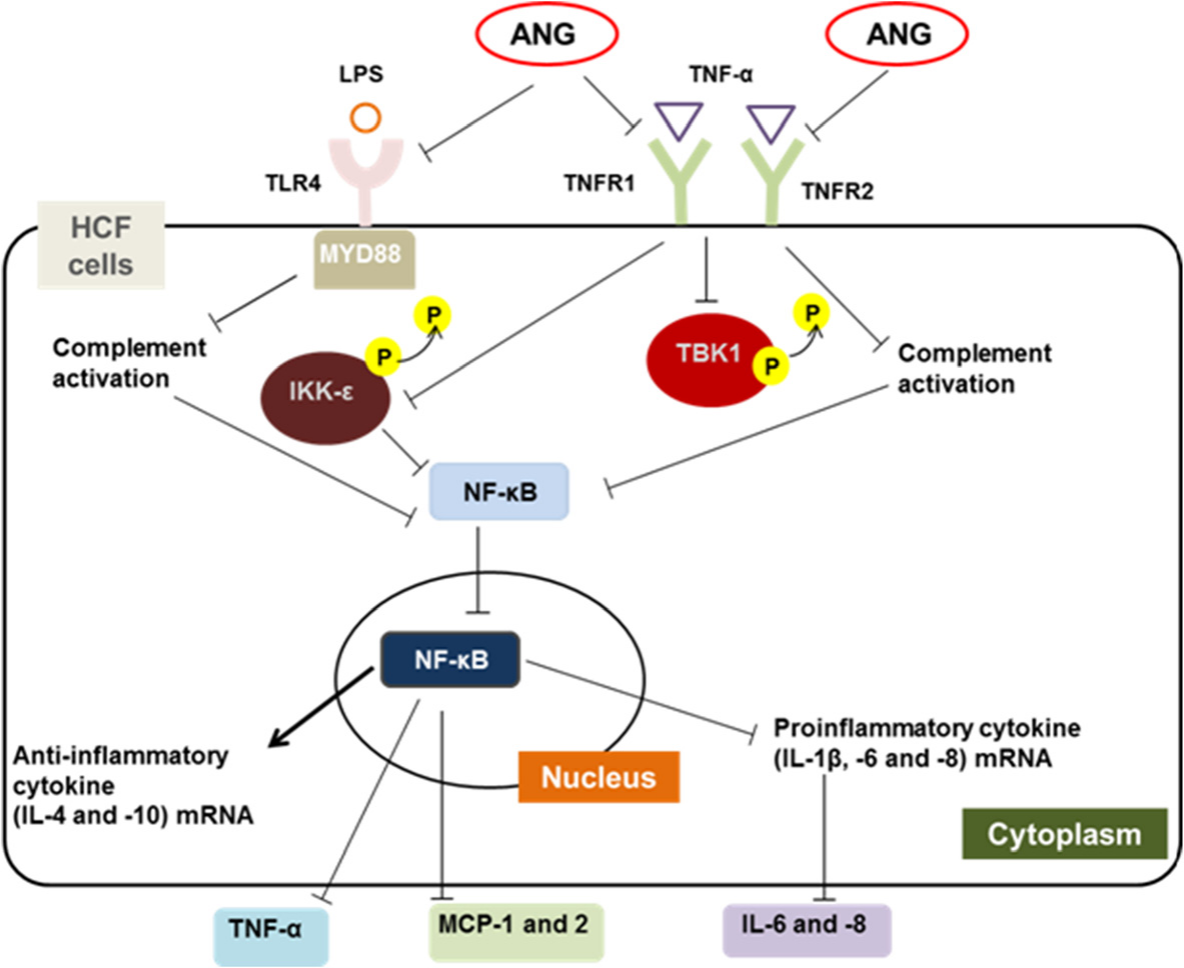
Schematic model illustrating the signaling pathway by which angiogenin (ANG) reduces the inflammatory responses involving TANK-binding kinase (TBK)1- and IkappaB kinase-epsilon (IKK-ε)-mediated nuclear translocation of nuclear factor-kappaB (NF-κB) in tumor necrosis factor-alpha (TNF-α)- or lipopolysaccharide (LPS)-induced inflammatory human corneal fibroblasts (HCFs). ANG down-regulates the mRNA expression of pro-inflammatory cytokines (interleukin [IL]-1β, IL-6 and IL-8) and up-regulates the mRNA expression of anti-inflammatory cytokines (IL-4 and IL-10). ANG suppresses the nuclear translocation of NF-κB through the inhibition of TNF-α receptor (TNFR)1 and TNFR2 mRNA expression and TBK1 production in TNF-α-induced inflammatory HCFs. ANG also reduces NF-κB nuclear translocation through a reduction in toll-like receptor (TLR)4 and myeloid differentiation primary response gene (MYD)88 mRNA expression and IKK-ε expression in LPS-induced inflammatory HCFs. The cascade underlying the effect of ANG results in a suppression of inflammatory cytokines and chemokines such as TNF-α, monocyte chemotactic protein (MCP)-1, MCP-2, IL-6 and IL-8

The schematic diagram illustrating the anti-inflammatory signaling pathways induced by ANG in TNF-α- or LPS-inflamed HCFs is shown in Fig. 8. Inflammatory signal induced by TNF-α or LPS is triggered via TLR4, MYD88, TNFR1, and TNFR2. The inflammatory response induces TBK1 and IKK-ε activation and mediates nuclear translocation of NF-κB. The mRNA expression of IL-1β, IL-6 and IL-8 is reduced by ANG and ANG treatment increases the mRNA expression of IL-4 and IL-10. ANG also inhibits the nuclear translocation of NF-κB through suppression of TBK1 and IKK-ε production. The anti-inflammatory effect induced by ANG eventually causes a decrease in the expression of pro-inflammatory cytokines and chemokines such as IL-6, IL-8, MCP-1, MCP-2, and TNF-α.

Although ANG treatment did not cause adverse effect such as injection on the ocular surface, in vivo animal studies conducted over a long term are required to further confirm the clinical application of ANG in corneal inflammation to eliminate the doubt of possible adverse effect. In a future study, the cytokine analysis to detect the reduction of pro-inflammatory cytokines in corneal stroma, tear fluids or aqueous humor and NF-κB suppression through the inhibition of IKK-ε may be required to confirm the anti-inflammatory activity of ANG in in vivo animal studies and to clarify the ANG-specific regulation of inflammation. Moreover, further study is needed to confirm ANG activity through comparison of anti-inflammatory effect between ANG and other drug such as steroids.

## Conclusion

The role of ANG in the inflammatory response has been little investigated. The finding of suppression of IKK-ε production by ANG in HCFs established in this investigation indicates that ANG can reduce the immune responses and corneal inflammation. Our results demonstrated that ANG down-regulated the inflammatory response triggered by NF-κB activation via inhibition of IKK-ε expression in TNF-α- or LPS-induced inflammatory HCFs. This study showing the anti-inflammatory effect of ANG proposes that ANG has a novel therapeutic potential against corneal inflammation.

## Ethics and consent to participate

The study protocol was reviewed and approved by the Institutional Review Board of Chung-Ang University Hospital (No. C2014849(1245)). Animal procedures in this study were performed in accordance with ARVO Statement for the Use of Animals in Ophthalmic and Vision Research. All procedures were performed according to the tenets of the Declaration of Helsinki, and informed consent was obtained for the use of human corneal tissues.

## Consent to publish

Not applicable.

## Availability of data and materials

All the data supporting the findings was contained within the manuscript.

## Abbreviations

ANG: angiogenin
HCF: human corneal fibroblast
IKK-ε: I kappa B kinase-epsilon
IL: interleukin
LPS: lipopolysaccharide
MCP: monocyte chemotactic protein
MYD: myeloid differentiation primary response gene
PCR: polymerase chain reaction
TBK: TANK-binding kinase.
TLR: toll-like receptor
TNF: Tumor necrosis factor
TNFR: TNF-alpha receptor
